# Remote ischemic preconditioning versus sham-control for prevention of anastomotic leakage after resection for rectal cancer (RIPAL trial): a pilot randomized controlled, triple-blinded monocenter trial

**DOI:** 10.1007/s00384-024-04637-4

**Published:** 2024-05-03

**Authors:** Julia Hardt, Steffen Seyfried, Hannah Brodrecht, Leila Khalil, Sylvia Büttner, Florian Herrle, Christoph Reissfelder, Nuh N. Rahbari

**Affiliations:** 1 Department of Surgery, Universitätsmedizin Mannheim, Medical Faculty Mannheim, Heidelberg University, Theodor-Kutzer-Ufer 1-3, 68167 Mannheim, Germany; 2 Medical Statistics, Biomathematics and Information Processing, Medical Faculty Mannheim, Heidelberg University, Mannheim, Germany

**Keywords:** Remote ischemic preconditioning, Ischemia‒reperfusion injury, Anastomotic leakage, Rectal cancer, Rectal resection

## Abstract

**Purpose:**

Remote ischemic preconditioning (RIPC) reportedly reduces ischemia‒reperfusion injury (IRI) in various organ systems. In addition to tension and technical factors, ischemia is a common cause of anastomotic leakage (AL) after rectal resection. The aim of this pilot study was to investigate the potentially protective effect of RIPC on anastomotic healing and to determine the effect size to facilitate the development of a subsequent confirmatory trial.

**Materials and methods:**

Fifty-four patients with rectal cancer (RC) who underwent anterior resection were enrolled in this prospectively registered (DRKS0001894) pilot randomized controlled triple-blinded monocenter trial at the Department of Surgery, University Medicine Mannheim, Mannheim, Germany, between 10/12/2019 and 19/06/2022. The primary endpoint was AL within 30 days after surgery. The secondary endpoints were perioperative morbidity and mortality, reintervention, hospital stay, readmission and biomarkers of ischemia‒reperfusion injury (vascular endothelial growth factor, VEGF) and cell death (high mobility group box 1 protein, HMGB1). RIPC was induced through three 10-min cycles of alternating ischemia and reperfusion to the upper extremity.

**Results:**

Of the 207 patients assessed, 153 were excluded, leaving 54 patients to be randomized to the RIPC or the sham-RIPC arm (27 each per arm). The mean age was 61 years, and the majority of patients were male (37:17 (68.5:31.5%)). Most of the patients underwent surgery after neoadjuvant therapy (29/54 (53.7%)) for adenocarcinoma (52/54 (96.3%)). The primary endpoint, AL, occurred almost equally frequently in both arms (RIPC arm: 4/25 (16%), sham arm: 4/26 (15.4%), p = 1.000). The secondary outcomes were comparable except for a greater rate of reintervention in the sham arm (9 (6–12) vs. 3 (1–5), p = 0.034). The median duration of endoscopic vacuum therapy was shorter in the RIPC arm (10.5 (10–11) vs. 38 (24–39) days, p = 0.083), although the difference was not statistically significant.

**Conclusion:**

A clinically relevant protective effect of RIPC on anastomotic healing after rectal resection cannot be assumed on the basis of these data.

## Introduction

Rectal cancer (RC) is the third leading cause of cancer-related mortality globally, with an incidence of more than 150,000 cases and accounting for more than 50,000 deaths per year in the United States [1]. Within the multidisciplinary management of RC, anterior resection (AR) remains the cornerstone of curative treatment.

The most devastating complication after AR is anastomotic leakage (AL), which occurs in 15–20% of patients [2]. Except for a temporary, defunctioning ostomy, no strategy has proven effective in reducing AL after rectal resection. However, even with defunctioning ostomy, the AL rate remains relatively high; when routine endoscopy was used after rectal resection prior to discharge, our data confirmed an AL rate of 18% [3].

AL has a severe impact on patients’ short- and long-term outcomes and is the main cause of postoperative mortality after rectal surgery. Prospective data from the British Colorectal Cancer Audit revealed perioperative mortality rates of 10% and 2% in patients with and without AL, respectively (p = 0.014) [4]. Numerous studies have reported a detrimental impact of AL on local recurrence (LR), overall survival (OS) and cancer-specific survival (CSS) [5] as well as on the functional outcomes of RC patients [6].

Effective measures to prevent AL have not yet been introduced into clinical practice and present a persistently unmet medical need. Since ischemic damage is one of the most common causes of anastomotic dehiscence, along with inadequate mobilization and subsequent tension, as well as technical and patient factors, it appears justified to examine the protective potential of measures that have already proven successful in preventing ischemic damage.

Remote ischemic preconditioning (RIPC) is such a measure and differs from other preconditioning strategies because it is performed remotely from target organs. Short episodes of ischemia and reperfusion are induced by temporary arterial occlusion of a limb to release cytokines, which mediate protection from ischemic injury in organs of interest. Numerous preclinical studies have shown that RIPC has a protective effect against ischemia‒reperfusion injury (IRI) in various organ systems, including the liver, heart, brain, and kidney. Mechanistically, RIPC induces serotonin release from platelets, which stimulates VEGF (vascular endothelial growth factor) secretion to upregulate IL (interleukin) 10 and Mmp (matrix metalloproteinase) 8 in target organs [7]. Several multicenter trials have provided level I evidence that RIPC reduces myocardial infarction [8], kidney injury and the need for renal replacement therapy [9] after elective aortic aneurysm repair and coronary bypass surgery.

This evidence, together with preclinical data indicating the protective effects of RIPC on the intestinal mucosa [10], justifies its clinical translation to the field of rectal cancer surgery. Therefore, the present study was designed to demonstrate the protective effect of RIPC on anastomotic healing after anterior resection for rectal cancer patients. The pilot RCT design was chosen because there is insufficient evidence to assess the impact of RIPC in this context. Therefore, a pilot RCT was needed to determine the effect size so that a confirmatory RCT could be planned on this basis.

## Methods

This study was designed in accordance with the Declaration of Helsinki. After approval from the institutional ethics committee (2019-730 N) and prospective registration in the German Clinical Trials Register (DRKS00018942), the study was conducted as a pilot randomized controlled, triple-blind, monocenter trial at the Department of Surgery at the University Hospital in Mannheim, Germany between 10/12/2019 and 19/06/2022. Adult patients with histologically proven rectal cancer planned for elective anterior resection with primary anastomosis and without contraindications for the study intervention (such as peripheral arterial disease, infections or wounds on the upper extremity, poorly controlled diabetes mellitus or upper limb deep vein thrombosis) were eligible for inclusion in the study. Another exclusion criterion was the inability to provide informed consent. The screening for potential enrollment took place during preadmission consultations.

All operations and perioperative management were embedded in the context of an ERAS^®^ (enhanced recovery after surgery) clinical pathway for colorectal resection. As of March 2021, the Department of Surgery at the University Hospital in Mannheim, Germany, was an ERAS^®^ Center certified by the ERAS^®^ Society.

This randomized trial is reported in line with the CONSORT (Consolidated Standards of Reporting Trials) statement [11]. A CONSORT checklist is provided as an online supplementary document.

### Sample size calculation

According to the explorative study design, a case number calculation was not necessary. The number of evaluable patients per group was 25. Evaluable patients were those who met the criteria for evaluability according to intention-to-treat (ITT) analysis and per-protocol (PP) analysis. Patients included in the study according to the ITT analysis set were those who received either RIPC or sham-RIPC (control) perioperatively. Patients treated according to the protocol for the per-protocol analysis were those who participated in the study after study inclusion up to and including the last study visit and underwent the associated investigations according to the protocol.

To compensate for a dropout rate of approximately 10%, a total of 56 patients, comprising both groups, were initially planned. After the dropout rate was lower than expected, recruitment could be terminated after only 54 patients.

### Randomization and blinding

Patients were randomized 1:1 immediately before the induction of anesthesia. The randomization sequence was computer generated using the statistical software R (https://www.r-project.org/) and concealed from the investigators. Patients, surgeons, endoscopists, outcome assessors, and any staff providing care to the patients were blinded regarding treatment allocation.

### Study intervention

In RIPC, a blood pressure cuff was placed around an arm immediately prior to surgery (after induction of anesthesia and before incision) and inflated to 200 mmHg or a pressure ≥ 50 mmHg above the systolic pressure for five minutes. This corresponded to the ischemic stimulus being distant from the target organ and was followed by a five-minute break. The entire procedure was performed three times for a total of three ten-minute cycles (for a total of 30 min per patient). In the sham arm, a blood pressure cuff was placed around the upper extremity, but the cuff was not inflated.

### Blood sampling and biomarker analysis

VEGF, a mediator of the effect of RIPC, and the cell death biomarker HMGB1 were measured in blood serum samples collected at three different time points: immediately before the start of RIPC/sham-RIPC, immediately after the complete RIPC/sham-RIPC procedure and three hours after RIPC.

VEGF (R&D Systems, Minneapolis, MN) and HMGB1 (Novusbio/Bio-Techne GmbH, Wiesbaden, Germany) levels were determined in duplicate by enzyme-linked immunosorbent assay (ELISA) following the manufacturer’s instructions.

### Surgical procedures

All patients scheduled for elective anterior resection with primary anastomosis were eligible for inclusion in the study, regardless of the choice of surgical approach (laparoscopic, robotic-assisted, or open). All resections included partial (PME) or total mesorectal excision (TME) depending on the tumor location in the rectum. Reconstruction was routinely performed by creating a side-to-end anastomosis using the EEA™ 28 circular stapler with Tri-Staple™ technology (Medtronic, Minneapolis, MN, USA). The standard reconstruction technique after intersphincteric resection for ultralow rectal cancer patients is end-to-end handsewn coloanal anastomosis. For the robotic approach, the DaVinci Xi^®^ or X^®^ systems (Intuitive Surgical Inc., Sunnyvale, CA, USA) were used.

### Outcomes

The primary endpoint was AL within 30 days after surgery. According to the definition of the International Study Group of Rectal Cancer, AL is defined as a defect of the intestinal wall in the area of the anastomosis (including (staple) sutures of a potentially created neorectal reservoir) that leads to intra- and extraluminal compartments communicating with each other. The degree of severity was also categorized according to the classification of the International Study Group of Rectal Cancer: AL grade A - no deviation from the planned clinical course, grade B - reintervention (e.g., endoluminal vacuum therapy, CT (computed tomography)-guided abscess drainage) required but no relaparotomy, grade C - relaparotomy required [12].

In patients with clinical symptoms (pain, fever, elevated infectious parameters, tachycardia/hypotension), AL was confirmed by endoscopic or radiologic (computed tomography scan with rectal contrast) investigations. Asymptomatic patients were evaluated on a routine basis by endoscopy on postoperative day (POD) 5 ± 1.

Secondary endpoints included perioperative morbidity and mortality (Clavien–Dindo classification), necessity/duration of reinterventions (endoluminal vacuum therapy, interventional drainage, reoperation), length of hospital/intensive care unit stay and readmission.

The effects of RIPC on biomarkers of IRI (VEGF) and necrotic cell death (HMGB1) were measured in serum before the study intervention (t0), immediately after (t1), and after another 3 h (t2) using ELISA.

### Statistical analysis

The absolute and relative frequencies were quoted for qualitative parameters. The mean and standard deviation were calculated for normally distributed quantitative variables, while the median and range are given for skewed parameters. To identify a relationship between two qualitative parameters, the chi-square test or, if necessary, Fisher's exact test was used. To compare two groups with regard to quantitative variables, two-sample t tests were used for normally distributed data, and Mann‒Whitney U tests were used for skewed data.

All the statistical calculations were performed using SAS software, release 9.4 (SAS Institute, Inc., Cary, NC, USA). In general, a test result was considered statistically significant for p ≤ 0.05.

## Results

**Fig. 1:**
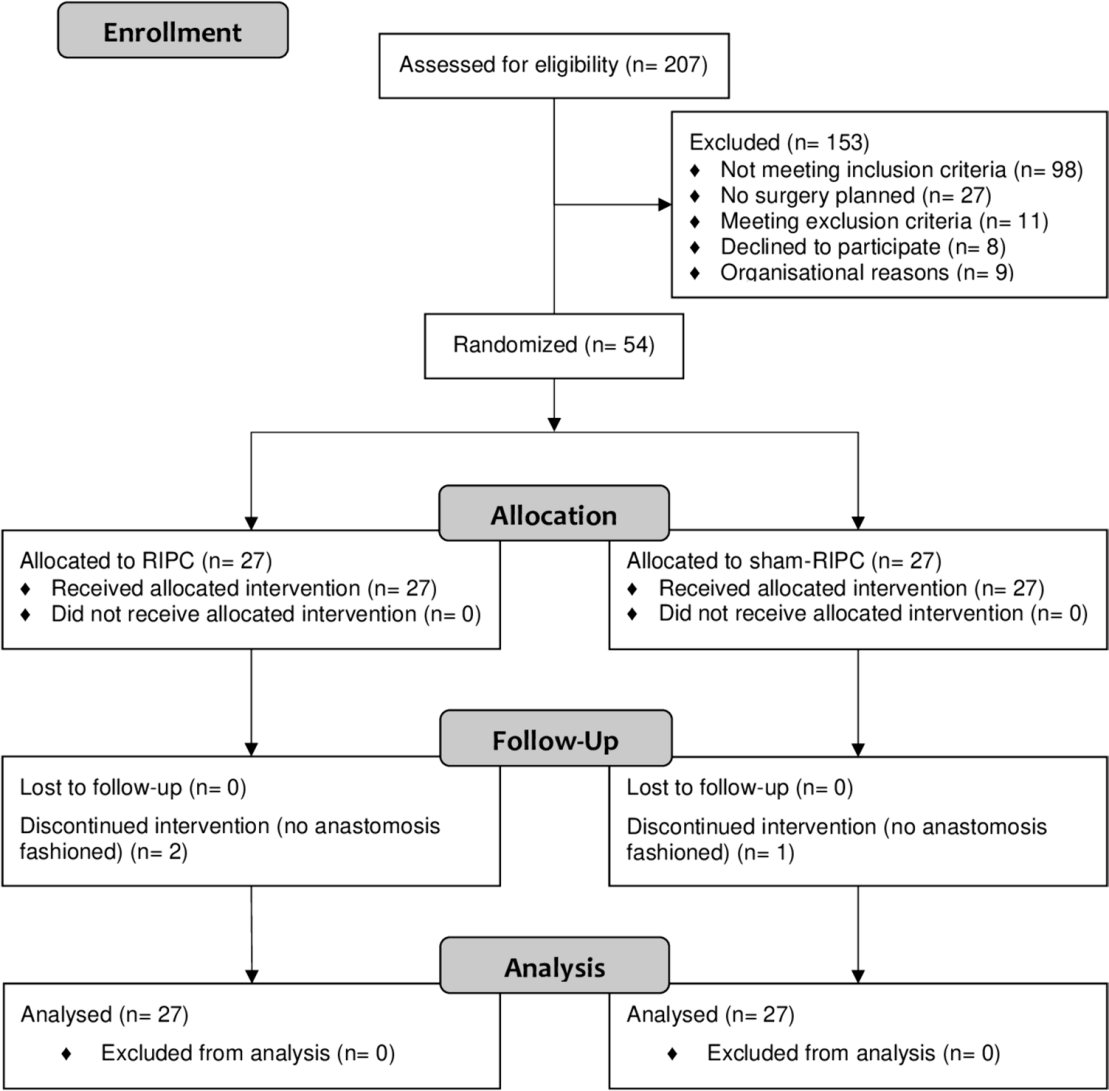
CONSORT (Consolidated Standards of Reporting Trials) flow diagram of the progress of the two study arms through the phases of the trial (based on the updated CONSORT guidelines on reporting parallel group randomized trials: http://www.consort-statement.org/consort-statement/flow-diagram)

From December 2019 to June 2022, 54 patients at the University Medical Center Mannheim were included in the study and randomized (27 per arm). Figure 1 presents the flow diagram according to the CONSORT statement.

Patients’ baseline characteristics were equally distributed in both study arms (Table 1) apart from a surplus of men in the RIPC arm (22:5 (81.5%:18.5%) vs. 15:12 (55.6%:44.4%), p = 0.043). The mean age was 61.28 ± 12.7 years, and the mean body mass index (BMI) was 26.45 ± 5.31 kg/m^2^. According to the ASA Physical Status Classification System, more than two-thirds of the patients were classified as ASA (American Society of Anesthesiologists) II (38/54 (70.4%)), 9/54 (16.7%) were classified as ASA III, and 7/54 (13%) were classified as ASA class I. The median serum albumin concentration measured during the last preoperative check-up was in the middle of the normal range (39 (20–46) g/l). Adenocarcinoma was by far the most common indication for surgery (52/54 (96.3%)), whereas only one patient (1.9%) underwent surgery for squamous cell carcinoma, and one (1.9%) underwent surgery for undifferentiated carcinoma of the rectum. Most of the patients had undergone neoadjuvant treatment, either as chemoradiation (20/54 (37%)) or chemotherapy (9/54 (16.7%)). Almost half of the tumors were located in the mid rectum (26/54 (48.2%)), followed by 17/54 (31.5%) in the lower third and 11/54 (20.4%) in the upper third of the rectum.

**Table 1:** Baseline characteristics of the study cohort

	**RIPC (n = 27)**	**Sham (n = 27)**	**Overall Cohort** **(n = 54)**	**p**
**Age** [years]	58.85 ± 11.8	63.7 ± 13.32	61.28 ± 12.7	0.163
**Gender** (male:female)	22:5 (81.5%:18.5%)	15:12 (55.6%:44.4%)	37:17 (68.5%:31.5%)	0.043
**BMI** [kg/m^2^]	27.47 ± 5.3	25.42 ± 5.22	26.45 ± 5.31	0.157
**Preoperative serum albumin** [g/l]*	38.55 (20–44)	40 (21–46)	39 (20–46)	0.314
**ASA**				1.000
I	4 (14.8%)	3 (11.1%)	7 (13%)	
II	19 (70.4%)	19 (70.4%)	38 (70.4%)	
III	4 (14.8%)	5 (18.5%)	9 (16.7%)	
**Diabetes mellitus**				0.275
Not insulin-dependent	2 (7.4%)	3 (11.1%)	5 (9.3%)	
Insulin dependent	3 (11.1%)	0	3 (5.6%)	
**Arterial hypertension**	15 (55.6%)	11 (40.7%)	26 (48.2%)	0.276
**Nicotine abuse**	11 (40.7%)	7 (25.9%)	18 (33.3%)	0.248
Pack years	30 (18–111)	30 (2–100)	30 (2–111)	0.855
**Alcohol abuse**	2 (7.4%)	2 (7.4%)	4 (7.4%)	1.000
**Neoadjuvant therapy**				1.000
Chemotherapy	4 (14.8%)	5 (18.5%)	9 (16.7%)	
Chemoradiation	10 (37%)	10 (37%)	20 (37%)	
**Tumor histology**				0.491
Adenocarcinoma	25 (92.6%)	27 (100%)	52 (96.3%)	
Squamous cell carcinoma	1 (3.7%)	0	1 (1.9%)	
Undifferentiated	1 (3.7%)	0	1 (1.9%)	
**Tumor location**				0.229
Upper third	6 (22.2%)	5 (18.5%)	11 (20.4%)	
Mid third	10 (37%)	16 (59.3%)	26 (48.2%)	
Lower third	11 (40.7%)	6 (22.2%)	17 (31.5%)	
**TNM classification**				
pT category				0.730
T0	3 (11.1%)	2 (7.4%)	5 (9.3%)	
T1	1 (3.7%)	2 (7.4%)	3 (5.6%)	
T2	12 (44.4%)	10 (37%)	22 (40.7%)	
T3	9 (33.3%)	13 (48.2%)	22 (40.7%)	
T4	1 (3.7%)	0	1 (1.9%)	
T4b	1 (3.7%)	0	1 (1.9%)	
pN category				0.369
N0	19 (70.4%)	19 (70.4%)	38 (70.4%)	
N1a	3 (11.1%)	1 (3.7%)	4 (7.4%)	
N1b	4 (14.8%)	2 (7.4%)	6 (11.1%)	
N1c	0	3 (11.1%)	3 (5.6%)	
N2b	1 (3.7%)	2 (7.4%)	3 (5.6%)	
Grading				1.000
G1	4 (14.8%)	3 (11.1%)	7 (13%)	
G2	22 (81.5%)	23 (85.2%)	45 (83.3%)	
GX	1 (3.7%)	1 (3.7%)	2 (3.7%)	
Resection status				0.236
R0	24 (88.9%)	27 (100%)	51 (94.4%)	
R1	3 (11.1%)	0	3 (5.6%)	
M category				0.511
M0	25 (92.6%)	23 (85.2%)	48 (88.9%)	
M1	0	2 (7.4%)	2 (3.7%)	
M1a	1 (3.7%)	2 (7.4%)	3 (5.6%)	
M1b	1 (3.7%)	0	1 (1.9%)	
**UICC stage**				1.000
0	2 (7.4%)	2 (7.4%)	4 (7.4%)	
I	12 (44.4%)	11 (40.7%)	23 (42.6%)	
IIA/B	5 (18.5%)	6 (22.2%)	11 (20.4%)	
IIIA	2 (7.4%)	1 (3.7%)	3 (5.6%)	
IIIB	4 (14.8%)	4 (14.8%)	8 (14.8%)	
IIIC	0	1 (3.7%)	1 (1.9%)	
IV	2 (7.4%)	2 (7.4%)	4 (7.4%)	

Number (proportion in %); median (minimum-maximum); mean ± standard deviation

*ASA* Classification of American Society of Anesthesiologists, *BMI* body mass index, *RIPC* remote ischemic preconditioning, *UICC* Union for international cancer control

*Missing data: Preoperative serum albumin n = 1 (RIPC)

The intraoperative and surgical technical characteristics did not differ between the two study arms (Table 2). Propofol—for anesthesia induction—was administered to the majority of patients (44/54 (84.6%)), but only 8/54 (15.4%) patients received continued propofol for anesthesia maintenance. Rectal resection was performed according to our institutional standards using minimally invasive techniques. More than half of the patients underwent a conventional laparoscopic abdominal approach (28/54 (51.9%)), and more than one-third underwent robot-assisted laparoscopy (19/54 (35.2%)). Laparotomy was performed in only 7/54 (13%) patients. Among the surgical procedures, low anterior resection (LAR) was by far the most common (47/54 (87%)). Multivisceral resection was the second most common treatment for organ-transcending tumors (5/54 (9.3%)), followed by anterior resection with PME (4/54 (7.4%)) and abdominoperineal resection (3/54 (5.6%)). The average operating time was 368.7 ± 131.17 min. In 90% of the patients (47/54), a protective loop ileostomy was performed. In the majority of patients, the anastomosis was created side-to-end via circular stapling (40/54 (74%)). Less common were transverse coloplasty with hand-sewn anastomosis (5/54 (9%)) or circular stapling (1/54 (1.9%)) and end-to-end anastomoses using either a circular stapler (3/54 (5.6%)) or a hand-sewn (2/54 (3.7%)). The median intraoperative blood loss was 250 (0–2000) millilitres.

**Table 2:** Surgical procedures and intraoperative outcomes

	**RIPC (n = 27)**	**Sham (n = 27)**	**Overall Cohort (n = 54)**	**p**
**Surgical procedure**				0.503
Low anterior resection + TME	22 (81.5%)	25 (92.6%)	47 (87%)	
Anterior resection + PME	3 (11.1%)	1 (3.7%)	4 (7.4%)	
Abdominoperineal resection	2 (7.4%)	1 (3.7%)	3 (5.6%)	
Multivisceral resection	4 (14.8%)	1 (3.7%)	5 (9.3%)	0.351
**Propofol application**	26 (96.3%)	26 (96.3%)	52 (96.3%)	1.000
Only for induction of anesthesia	22 (84.6%)	22 (84.6%)	44 (84.6%)	
Also for anesthesia maintenance	4 (15.4%)	4 (15.4%)	8 (15.4%)	
**Surgical approach**				0.483
Robotic	8 (29.6%)	11 (40.7%)	19 (35.2%)	
Laparoscopic	14 (51.9%)	14 (51.9%)	28 (51.9%)	
Open	5 (18.5%)	2 (7.4%)	7 (13%)	
**Duration of surgery** [minutes]	374.11 ± 131.55	363.3 ± 133.06	368.7 ± 131.17	0.562
Protective ileostomy*	24 (92.3%)	23 (88.5%)	47 (90.4%)	1.000
**Anastomosis**				0.530
Side-to-end via circular stapling	17 (63%)	23 (85%)	40 (74%)	
End-to-end via circular stapling	2 (7.4%)	1 (3.7%)	3 (5.6%)	
End-to-end hand sewn	1 (3.7%)	1 (3.7%)	2 (3.7%)	
Transverse coloplasty with hand sewn anastomosis	4 (15%)	1 (3.7%)	5 (9.3%)	
Transverse coloplasty with circular stapling	1 (3.7%)	0	1 (1.9%)	
No anastomosis fashioned	2 (7.4%)	1 (3.7%)	3 (5.6%)	
**Blood loss** [ml]	300 (0–1500)	200 (0–2000)	250 (0–2000)	0.229

Number (proportion in %); median (minimum-maximum); mean ± standard deviation

*PME* partial mesorectal excision, *RIPC* remote ischemic preconditioning, *TME* total mesorectal excision

*Missing data: Protective ileostomy n = 2 (RIPC: n = 1, Sham: n = 1)

RIPC could be performed in all patients without any complications, and there were no adverse events associated with the study intervention. There were three protocol deviations throughout the course of the study: two patients in the RIPC arm and one patient in the sham arm received no anastomosis but underwent an abdominoperineal resection against preoperative expectations and thus formally did not meet the inclusion criterion of a rectal resection with primary anastomosis.

The results regarding the predefined endpoints of the study are outlined in Table 3. The primary endpoint AL within 30 days after surgery did not significantly differ between the two arms (RIPC arm: 4/25 (16%) vs. sham-control arm: 4/26 (15.4%), p = 1.000), and the severity of AL (grades A-C according to the classification of the International Study Group of Rectal Cancer) was also similarly distributed (grade A: one patient each in both arms, grade B: two patients in the RIPC arm and three patients in the sham-control arm; grade C: only one patient in the RIPC arm). The median time interval between surgery and AL diagnosis was 6 (4–16) days, with no significant difference between the two study arms (RIPC arm: 5 (4–12) days, sham-control arm: 6 (5–16) days), p = 0.381). Two patients in each arm underwent endoscopic vacuum therapy for treatment of their grade B AL. The duration of healing of AL measured by the duration of endoscopic vacuum therapy was shorter in the experimental arm (median duration of endoscopic vacuum-assisted closure: RIPC arm: 10.5 (10–11) vs. sham-control arm: 38 (24–39) days). However, this difference was not statistically significant (p = 0.083).

**Table 3:** Primary and secondary outcomes

	**RIPC (n = 27**)	**Sham (n = 27)**	**Overall Cohort (n = 54)**	**p**
*No anastomosis fashioned*	*2 (7.4%)*	*1 (3.7%)*	*3 (5.6%)*	
**Primary outcome**				
Anastomotic leakage	4 (16%)	4 (15.4%)	8 (15.7%)	1.000
Time interval until AL diagnosis [days]	5 (4–12)	6 (5–16)	6 (4–16)	0.381
Severity of anastomotic leakage**				1.000
Grade A	1 (4%)	1 (4%)	2 (3.9%)	
Grade B	2 (8%)	3 (11.5%)	5 (9.8%)	
Grade C	1 (4%)	0 (0%)	1 (4%)	
**Reintervention**				
Abdominal reintervention required	4 (14.8%)	3 (11.1%)	7 (13%)	1.000
Number of reinterventions per patient	3 (1–5)	9 (6–12)	5 (1–12)	0.034
Type of reintervention				
Percutaneous drainage	1 (3.7%)	0	1 (1.9%)	
Percutaneous drainage and endoscopic vacuum therapy	0	1 (3.7%)	1 (1.9%)	
Endoscopic vacuum therapy	2 (7.4%)	2 (7.4%)	4 (7.4%)	
Duration [days]	10.5 (10–11)	38 (24–39)	24 (10–39)	0.083
Other	1 (3.7%)	0	1 (1.9%)	
**Abdominal revision**	1 (3.7%)	1 (3.7%)	2 (3.7%)	1.000
**Postoperative hospital stay**				
Length of postoperative hospital stay [days]	7 (4–29)	7 (4–49)	7 (4–49)	0.951
Intensive care required	2 (7.4%)	0	2 (3.7%)	
Length of intensive care [days]	1	0	1	
**Readmission**	3 (11.1%)	3 (11.1%)	6 (11.1%)	1.000
**Postoperative 30-day morbidity (Clavien-Dindo)**				
Grade I and II	13 (48.1%)	7 (25.9%)	20 (37%)	0.167
Grade IIIa	4 (14.8%)	2 (7.4%)	6 (11.1%)	
Grade IIIb	2 (7.4%)	2 (7.4%)	4 (7.4%)	
Grade IV and V	0	0	0	
≥ Grade III	6 (22.2%)	4 (14.8%)	10 (18.5%)	0.088
**HMGB1** [ng/ml]*				
t0	88 (21–185)	90 (22–132)	89 (21–185)	0.763
t1	105 (25–235)	92 (29–156)	94 (25–235)	0.367
t2	84 (21–182)	71 (18–154)	76 (18–182)	0.123
**VEGF** [pg/ml]*				
t0	334 (98–1203)	284 (71–922)	308 (71–1203)	0.187
t1	342 (81–1112)	277 (74–998)	311 (74–1112)	0.357
t2	362 (94–1538)	330 (94–1170)	357 (94–1538)	0.367

Number (proportion in %); median (minimum-maximum); mean ± standard deviation

*AL* anastomotic leakage *HMGB1* High-Mobility Group Box 1, *RIPC* remote ischemic preconditioning, *VEGF* vascular endothelial growth factor

*Missing data: HMBG1 t0 n = 3 (RIPC: n = 2, Sham: n = 1), HMGB1 t1 and t2 n = 4 (RIPC: n = 2, Sham: n = 2), VEGF t0 n = 3 (RIPC: n = 2, Sham: n = 1), VEGF t1 and t2 n = 4 (RIPC: n = 2, Sham: n = 2)

**Severity of anastomotic leakage classified in Grade A (no symptoms), grade B (interventional treatment), grade C (reoperation) [12]

**Fig. 2:**
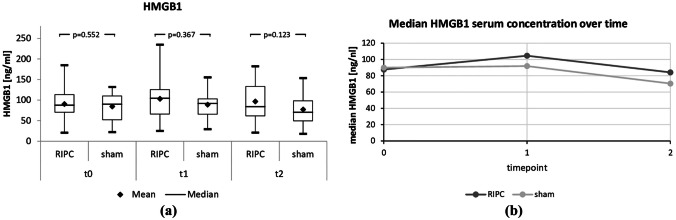
Box plots (**a**) and line diagram (**b**) of serum HMGB1, a biomarker of cell death, at three different measurement times, t0, t1 and t2 (immediately before the start of RIPC, immediately after the complete RIPC procedure, and 3 h after the end of RIPC)

**Fig. 3:**
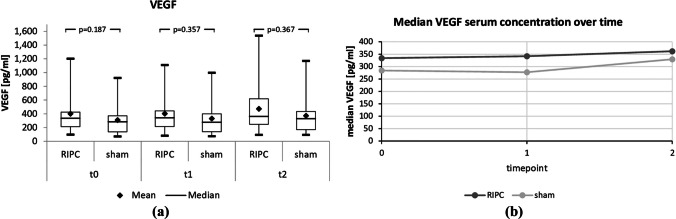
Box plots (**a**) and line diagram (**b**) of serum VEGF, a biomarker of ischemia‒reperfusion injury and RIPC-induced regulatory circuitry, at three different measurement times, t0, t1 and t2 (immediately before the start of RIPC, immediately after the complete RIPC procedure, and 3 h after the end of RIPC)

As shown in Table 3 and Figs. 2 and 3(a and b), there were no significant differences between the two arms in terms of HMGB1 and VEGF at any of the three time points studied.

The clinical secondary outcomes were comparable between the two arms, except for a significantly greater number of reinterventions per patient in the sham arm (9 (6–12) vs. 3 (1–5), p = 0.034) due to the fact that secondary anastomotic healing took significantly longer under endoscopic vacuum therapy and therefore more endoscopic sponge changes were required in these patients. In detail, the following reinterventions were necessary: In the RIPC arm, one patient received CT-guided abdominal drainage, two patients received endoscopic vacuum therapy and one patient developed severe postoperative paralytic ileus and therefore had to have a nasogastric tube inserted. In the sham arm, one patient received both CT-guided abdominal drainage and endoscopic vacuum therapy, whereas two patients only required endoscopic vacuum therapy. There were more overall complications in the RIPC arm; however, major morbidity was comparable (grade III: 6/27 (22.2%) in the RIPC arm, 4/27 (14.8%) in the sham-control arm, p = 0.117). None of the patients experienced grade IV or V complications.

There were two abdominal reoperations in the entire study cohort (one in each arm, p = 1.000). One patient in the RIPC arm required revision surgery for grade C AL on POD 22 after laparoscopic intersphincteric resection. During the revision operation, the left hemicolon leading down to the anastomosis was found to be ischemic. Therefore, the left hemicolon and the remaining rectum were resected, and a terminal colostomy was placed. Moreover, one patient in the control arm underwent revisional surgery with ileostomy reversal on POD 9 for recurrent episodes of mechanical ileus due to torsion of the protective ileostomy.

Postoperative hospital stays were well balanced in both arms (7 (4–29) days in the RIPC arm and 7 (4–49) days in the sham-control arm, p = 0.951). Only two patients (both from the RIPC arm) required intensive care, but only for one day each. The indications for admission to the intensive care unit were a prolonged need for intubation due to pronounced facial swelling after more than eight hours of surgery and monitoring after a long operation and in the case of relevant comorbidities.

Three patients per arm had to be readmitted. Two patients from the RIPC arm were readmitted on POD 24 and 25 after laparoscopic low anterior resection due to the subileus, which could be successfully treated conservatively in both patients. Moreover, one patient from the sham-control arm presented on POD 30 via the emergency room with unexplained sepsis. CT revealed COVID-19 pneumonia, which is why the patient was admitted to the internal medicine intensive care unit.

## Discussion

This is the first randomized controlled trial to compare RIPC versus a sham control for the prevention of AL after rectal cancer surgery. Within the 30-day follow-up, there was no difference in the AL rate between the two arms. The overall AL rate of 15.7% aligns well with the literature [13]. However, it must also be taken into account that the AL rate in this study was highly sensitive. All patients underwent control endoscopy of the anastomosis on POD 5 ± 1 in the course of the study. Thus, even small suture dehiscences were detected that had not become clinically apparent. Since the evidence on AL after rectal resection often comes from studies that only detected symptomatic AL, it can be assumed that the AL rate in our RCT appears to be rather high since without routine endoscopy, approximately one-fourth of the AL cases would probably have remained undetected.

Interestingly, the duration of secondary anastomotic healing after the occurrence of AL, measured by the duration of endoscopic vacuum therapy, was shorter in the RIPC arm (median duration of endoscopic vacuum-assisted closure: 10.5 (10–11) days in the RIPC arm vs. 38 (24–39) days in the sham-control arm). Although this difference has not yet reached statistical significance, one could still speculate whether RIPC has a certain supportive effect on anastomosis healing. It could also be hypothesized that RIPC may need to be performed over a longer period of time or more frequently to produce clinically significant effects on anastomotic healing. However, this cannot be inferred in any way on the basis of our data, and there are also no results from the literature.

Evidence regarding the preventive effect of RIPC on intestinal ischemic damage is sparse and comes mainly from cell culture and animal models. In these studies, intestinal damage was predominantly measured by histological scores or an increase in markers of cell damage (e.g., lactate dehydrogenase (LDH)). A study by Holzner et al. on anastomotic healing of the small intestine in a rat model showed heterogeneous results: although mucosal damage was reduced, there was no improvement in anastomotic stability [14]. Concordant results from the rat model were also published by Hummitzsch et al. [10]. The evidence from clinical trials consists of a handful of monocenter randomized trials with small sample sizes and heterogeneous results. Li et al. investigated the effect of RIPC on intestinal and pulmonary injury in 62 patients who underwent open infrarenal abdominal aortic aneurysm repair and reported that RIPC significantly attenuated intestinal and pulmonary damage [15]. In contrast, Struck et al. detected no impact of RIPC on intestinal injury, as measured by an increase in intestinal fatty acid binding protein (I-FABP), in thirty patients undergoing cardiopulmonary bypass surgery [16].

In the present study, clinical secondary outcomes were comparable between both arms, except for a significantly greater rate of reintervention in the sham-control arm (9 (6–12) vs. 3 (1–5), p = 0.034), but with comparable major morbidity (≥ grade III). There are also very few data in the literature on the influence of RIPC on clinical outcomes after intestinal surgery. A recent RCT from China investigated the impact of RIPC on the recovery of bowel function in 80 patients undergoing elective laparoscopic surgery for colorectal cancer [17]. Yang et al. reported that RIPC neither enhanced the recovery of bowel function nor decreased the incidence of postoperative ileus. However, RIPC shortened the median time to stool and significantly decreased postoperative levels of tumor necrosis factor-α (TNF-α) and C-reactive protein (CRP).

Moreover, the present study could not confirm the previously described key mediating role of VEGF in the RIPC mechanism [7]. VEGF levels increased steadily after the (sham) intervention in both arms, but there was no significant difference between the two intervention arms. Our results thus contradict the data from Limani et al. who showed in an animal model that VEGF is a key mediator of protective RIPC effects in livers. The authors were not only able to prove that RIPC stimulates the serotonin-VEGF axis, but also that all protective RIPC effects are absent when a neutralizing VEGF antibody is used [18]. The reasons why RIPC failed to induce significant activation of the serotonin-VEGF axis in the present study remain unclear.

Since RIPC is known to reduce IRI-induced organ damage, among other mechanisms, by opening a potassium channel that inhibits the proinflammatory HMGB1 signaling pathway [19], we expected to observe decreased levels of HMGB1 after RIPC in comparison with those in the sham-control arm. However, our results failed to support the impact of RIPC on HMGB1: there was no significant difference between the two arms at any of the measurement time points. This is not consistent with the literature, at least in part. The proinflammatory cytokine HMGB1 plays a central role in a wide variety of organ damage, including that of ischemic or septic genesis. HMGB1 is known to activate the TLR4 signaling pathway which causes an increase in inflammatory processes. The data from Koh et al. from a mouse model study investigating the hepatoprotective effect of RIPC demonstrated that RIPC was able to downregulate HMGB1 with a consecutive anti-inflammatory and hepatoprotective effect [19]. Similar effects are also described by Limani et al.: in their mouse model study comparing the protective effects of different preconditioning approaches in aged livers, RIPC significantly reduced serum HMGB1 levels [18]. Similar to the lack of RIPC-induced effects on VEGF, we can only speculate why our data are not consistent with the results of Koh and Limani. Perhaps the results from the animal model are not entirely transferable to the human model after all.

The significance of the study results is limited by several factors. First, this study is limited by its pilot trial design and small sample size. Second, the different nature of the operations in terms of the extent of resection and the height and technique of the anastomoses contributes to a certain degree of surgical heterogeneity, even if - as is to be expected in a randomized study - the distribution between the two arms was comparable. Third, the use of propofol for anesthesia induction (or maintenance) could have introduced confounders regarding the effects of RIPC because propofol may attenuate the protective effect of RIPC, as revealed by two multicenter trials, the ERICCA [20] and RIPHeart [21]. However, all the evidence on the inhibitory effect of propofol on RIPC is related to its cardioprotective effect. There is insufficient evidence regarding other organs involved. In addition, several studies have demonstrated the positive effects of RIPC on the lung and intestine despite the use of propofol [15].

In conclusion, based on the results of this randomized controlled trial, RIPC cannot be recommended as a routine measure for the prevention of AL after anterior resection for rectal cancer. Since this pilot study did not show a significant effect of the study intervention, no clear rationale for a definitive RCT can be derived from the results. However, the tendency toward faster secondary anastomotic healing under endoscopic vacuum therapy for Grade B AL in the RIPC arm could indicate at least a certain supportive effect and should be investigated in further clinical studies.

## Data Availability

The data that support the findings of this study are available from the corresponding author upon reasonable request.
